# Dental Hints for Country Medical Practitioners

**Published:** 1880-01

**Authors:** G. Newkirk

**Affiliations:** Wenona, Ill.


### Article IV.

### Dental Hints for Country Medical Practitioners. By G. Newkirk, m.d., Wenona, Ill.

### PART II.

*Examinations of Teeth.*—The first thing to attend to is the securing of good light. In examining the upper jaw, the rest should be so adjusted that the patient's head shall be thrown well back, the occiput on a line with or below the chin. For the lower jaw, the head should be about perpendicular with the body.

In the greater number of cases, the patient will point out correctly the location of the offending tooth, but not always. It is not safe to take his mere say-so as conclusive. There is no place in the body where reflex action is more apt to deceive than in connection with the nervous system of the teeth. A perfectly sound tooth may be pointed out as the offender, while the real source of trouble may exist at a point entirely unsuspected. Not long since a regular physician sent his son to me for the extraction of an upper molar that was almost perfectly sound. However, a second molar of the lower jaw, on the opposite side, had a large cavity, with a nearly exposed and tender pulp. Since an operation of capping and filling here, the boy has had no further annoyance in the region of the upper molar, nor with any of his teeth.

A tooth sensitive from a congested pulp, or the mere exposure of its dentine, will almost invariably respond to one or any of the following tests: A steel instrument touching or striking it; cold air or water ejected from the point of a syringe directly upon it; the introduction of a cold instrument into the cavity of decay, or pressure made in the direction of the pulp. These tests should always be applied with such care and gentleness as not to cause unnecessary pain.

With reference to the advisability of extractions the following points should be borne in mind. It is not always a sufficient apology for extraction of an aching or sensitive tooth that it belongs to the temporary set. As a rule, the temporary teeth ought to be preserved, if necessary by some sort of plastic filling, until near the time for the eruption of the permanent. They ought seldom to be removed until the root or roots have been in great part absorbed by the encroaching crowns of their successors to be. The country physician may often be of service to his little patients by using some sort of temporary protection for the temporary molars, in children from four to seven or eight years old, and sending them to a dentist for fillings sufficient to keep them useful and in place till the time proper for their removal.

Almost never should the physician extract a permanent incisor or canine for any young person; but if the troublesome tooth be a bicuspid, and the arch crowded so there seems to be not sufficient room for all the teeth, it may be for the permanent good of the others to have it removed. The same may be said of the first or sixth year molars, and more. If these teeth show indications of decay on all sides, appearing as they often times do to partake more of the nature of temporary than true permanent teeth, it is usually a safe course to remove them whenever they give trouble, and all the better if before the twelfth year, so that the second molars may erupt further forward and assist in closing the gap. If the removal becomes necessary, as it sometimes does, previous to the eruption of the second bicuspid, the space will be well filled, usually, by the time both this and the second molar have reached the line of articulation.

The preservation of a wisdom-tooth in the lower jaw is sometimes a matter of importance where a plate is to be worn above. A badly-decayed wisdom-tooth above is seldom fit for anything but extraction, and especially is this true where the first molar has been kept. But the middle molar should be seldom drawn by any one where there seems a possible chance of saving it, and the responsibility should be passed over to the dentist. If instead of crowded teeth there has been already the loss of some, the value of those remaining is, of course, proportionally increased, and our responsibility the greater in removing any of them. In such a case, we may observe whether the tooth in question has an antagonist in the opposite jaw; if not, and it is an upper tooth, it will probably never be of practical use for purposes of mastication. Natural teeth above are almost never used for articulation with artificial ones below, but lower teeth thus standing alone may become very valuable for use with artificial upper dentures, and should on no account be extracted where it is possible to avoid that alternative.

If the patient is wearing a plate and partial set of teeth, which he expects soon to have made over, or if he has lost so many of his teeth that the early wearing of a plate is inevitable, these may be so many arguments in favor of drawing a bad tooth upon the same jaw, and the physician, as well as the dentist, may the more readily yield under such circumstances, when solicited to extract.

Sometimes a patient applies to the physician with a troublesome root, and either a fully-developed or threatened alveolar abscess. If it be the root of a back tooth, the rule should be extraction, but not otherwise. A front root, however unpromising it may be in appearance, may be the basis for the insertion of a serviceable pivot tooth, or the fastening of an artificial crown. Present relief may be nearly always given by simply drilling through the pulp canal to the apex of the root, so giving vent to pus or confined gases, and the case may then be sent to the dentist.

The actual development of alveolar abscess, pointing from the root of any tooth, is quite certain evidence of the death of the pulp, in whole or in part, of that tooth. Almost invariably there has been death and decomposition of pulp and nerve substance, and the resulting fluid and gaseous matter has failed to find vent through the crown of the tooth, so seeks it by the ulcerative method through the process and gum. These conditions, when present, are usually the indirect result of caries. The actual or near exposure of the pulp, the sudden changes of temperature to which it has been subjected by cold air and hot and cold fluids have induced irritation, congestion, strangulation in the minute canals through which the blood supply must come, and death. They sometimes occur, however, independently of caries, not infrequently with elderly people whose teeth have been so worn down as to subject them, practically to the same vicissitudes. In all cases of this sort, instead of resorting to extraction, a free opening should be made into the pulp cavity, for present relief and further care of the tooth transferred to the dentist.

Whenever called on to look at any tooth, the opportunity should be improved for examining into the condition of the whole set and the necessary attention any may demand earnestly impressed on the mind of the patient. This may apply to filling, separating, regulating or cleaning. The country physician should also, like the dentist, ever impress upon his patients the duty of personal care and cleanliness with regard to the teeth. It may be here remarked, the important time for using the brush is just before retiring for the night, for the germs of decay are most often planted during the period of repose.

A "bad taste" in the mouth in the morning is more usually an indication of unhealthy changes that have taken place during the night, as a consequence of *uncleanliness*, than of "liver complaint" or indigestion, though this latter is often an associate.

He may very properly advise a change of diet for the benefit of children and young persons with teeth of poor structure. If wheaten grits, the so-called "Graham," corn and oat meal were more generally used by them the quality of their teeth would be much improved in consequence.

Let us consider next the mode of procedure necessary to the proper examination and *treatment* of a tooth.

You have your patient in a good light and proper position as hereinbefore mentioned, and we will suppose the tooth to be operated on is the right, lower, second molar. I take this for illustration because it is one often requiring attention, and is of great importance; furthermore, the saliva is here most apt to be troublesome, and, if we can succeed against it, we will hardly fail to do so elsewhere.

We will suppose the decay has begun from the face of the tooth. The size of the hole through the enamel is no sure index to the extent of decay. But there will be the opening more or less wide, bordered by the broken and irregular edge of enamel. Beyond this edge, for some distance probably, careful examination will reveal the extension of the color line, showing where decay has burrowed into the underlying dentine. You take your chisel and begin chipping away the enamel covering decay, and your first experience may be one of surprise at the ease with which it can be done. You cut away on either side of the fissure until the opening has been sufficiently enlarged for the easy introduction of an excavator. With this you proceed to remove the *débris* of dentine—whatever will readily yield—first in a direction toward the sides and not toward the pulp chamber. In working toward the latter, it will be safer for the amateur operator to use a burr. As you proceed, it will be necessary to occasionally syringe the cavity, to remove the chips and dust. Some dentists are cruel enough to use cold water for this purpose, even with sensitive teeth. You will be sure to have water about blood warm for this. You may have this very easily in any weather, by a simple contrivance. Let a tinsmith take a piece of heavy zinc or sheet iron 5x20 inches, round the corners, bend each end 5 inches down, at a right angle; tack this against any convenient upright, place a small night lamp on the lower shelf, a small tin cup on the upper, and you have an arrangement convenient for keeping water at the temperature you wish.

The pressure exerted by the mere swelling of the *débris*, or substances forced in from without in mastication, and held by the enamel shell, will often cause uneasiness or pain. The simple act of opening up the cavity, in such a case, by relieving the pressure that has been brought to bear on living dentine, or it may be the pulp itself, will give relief, and relief that may be made permanent by the use of proper protective means. The uneasiness gone or relived, the cavity may be soaked for a few minutes with the solution of morphia in creosote introduced on a bit of the cotton or spunk; then, that removed and the cavity thoroughly dried, a pellet of cotton saturated with sandarac varnish (or a gutta-percha pellet, if thought best, warmed over a lamp sufficiently to render it plastic) inserted, and the tooth dismissed to the dentist.

Care should be used in the application of the creosote and carbolic acid, that the pellet contains no excess; and where it is to be left in for a time the surface, after introduction, should be touched with a bit of dry spunk, so that there can be no overflow upon the soft parts.

In using the cotton and sandarac varnish, or other stopping, a care should be had not to press the pellet in too tight, and not to have it so large as to protrude beyond the margin of the cavity. Better a slight deficiency than too much material.

It will not be best, usually, for the physician to attempt the removal of all the decay, only that which can be brought away easily; that which is quite firm, especially over the pulp, had better be left to the superior skill and judgment of a competent dentist for retention or removal; for in some cases it is not best to remove *all* the decay, even for a permanent filling, but rather to *disinfect* and cover in with an air-tight stopping. All the physician need do in a case like this is to relieve pressure, and open up the way for the application of a soothing remedy and a temporary *dry* stopping. It is not often difficult to keep the saliva away so that a *dry* stopping may be made, by operating as follows: Have light muslin cut in squares of four to six inches; have one of these, or two together, folded in the form of a block about one by one and one-half inches, another in a small roll. The cavity has been sufficiently cleaned and disinfected; there is enough undercut to hold the filling you wish to introduce; the cotton and sandarac, or the gutta percha, are within easy reach—remember we are supposing the tooth to be on the right side below. Now, take the cotton block, and, with your pliers, place it against the inner surface of the tooth and alveolar ridge, and instruct the patient to hold it firmly against the gum with the index finger of the left hand. With the index finger of your own left hand you hold the roll between the ridge and cheek. You now have the tooth isolated, for the time being, from the secretions of the submaxillary and sublingual glands on the one hand and the buccal on the other. By these means you may make a dry operation even in a wet mouth, if you are not unreasonably slow. You have ready bits of spunk of proper size which you pick up one after another, insert and remove until they return dry, and then immediately introduce the stopping. On the left side below, the patient, with the same left forefinger, holds the *roll* between the process and *cheek*, and the operator the block between that and the tongue. In operating above only one roll is necessary placed between the ridge and cheek to protect from the flow of Steno's duct. The patient usually will not carry the tongue to an upper tooth when the mouth is well open, if he does he may be otherwise instructed. If the pulp is nearly or quite exposed, and the pain can be by no local means subdued, the use of creosote, morphia, clove oil, carbolic acid, hot applications, etc., all proving ineffectual, if the case be in a plethoric subject there may be a chance for relief in blood-letting or catharsis; if a nervous, the internal administration of one or two full doses of quinine, with morphia or Dover's powder, may be of speedy benefit. It must be borne in mind that occasionally the dental, like other nerves, are the seat of intrinsic pain, to be relieved like neuralgia elsewhere, by medical or surgical treatment.

Remember also this one necessity, always in operating on a very sensitive tooth, that every substance used in application shall be *warm*, neither much above nor any below blood heat. The instruments should be kept on a warm shelf or dipped in warm water before using, and the cotton or spunk, with the medicants, should be held over a lamp before being introduced. I speak of extreme cases.

If when all has been done it is evident that the tooth cannot be saved with a live pulp, shall you extract, or in common phrase, "kill the nerve?" becomes the question. I shall here state in brief what I believe to be a few plain facts and leave you to draw conclusions regarding pulpless teeth:

1. A large proportion of incisors, canines and bicuspids, teeth with single or double canals, may be saved for a time by an ordinarily skillful dentist, whether there is an alveolar abscess or not.

2. It will involve some patience, care and trouble on the part of patient as well as operator, and he ought to so understand it beforehand.

3. In some cases there will be a *good deal* of trouble, and possibly failure.

4. A pulpless tooth has not the same promise of long continuance and usefulness, as one in which the nerve tissue remains intact yet it may do well for many years, and front teeth especially should be given every chance of salvation.

5. The difficulties are increased and probabilities of success diminished as we go backward in the arch in our attempts to save pulpless teeth, and many people are unwilling to pay for the skill, time and trouble essential to success.

I have already expressed my aversion to the use of arsenic for purpose of destroying pulps, but if you chance to employ the agent there are some points not generally understood among doctors but very necessary to keep in remembrance, regarding its application.

It should be used in the form of a paste made with a little glycerine or mucilage, and best with the addition of a morphia salt. It should be applied by means of a small pellet of cotton directly to the pulp which must be quite exposed. The least particle will be sufficient if it touches the right spot. The pellet is to be carefully placed in position and kept there by another pellet of cotton and sandarac varnish, so that not any shall by any possibility escape from the cavity. After the application, the patient should go to the dentist within a few days.

As physicians, are familiar with the use of ether, you may if you will give a sufficient quantity, enter the pulp chamber with a sharp excavator or drill, remove the pulp with a suitable burr, cauterize the nerve stumps with carbolic acid, insert a loose pellet of cotton and send your patient home with instructions to go to a dentist within a week.

Where a cavity of decay causing trouble in a tooth is located on one of the proximal surfaces, it will often be difficult to give it proper attention without cutting away the semicircular piece of enamel over-lying it on the face of the tooth. Frequently the decay exists high enough up so that is an easy thing to do, the shell over it being quite thin. In doing this you will only anticipate the dentist, and give yourself light *into the affected part, and room to work.* A few words as to the *manner* of *handling instruments.* As a rule, dental instruments are to be held as one should properly hold a pen, between the thumb and the first and second fingers. The " Spencerian " position, wherein the second finger drops a little below so that the pen rests upon the edge of the nail, is the best for the complete control of any light instrument. It insures steady movement, accurate touch, and sufficient force for all ordinary work. Burrs and drills are to be held with the large end in the palm of the hand which exerts the necessary pressure, while the thumb and fingers revolve the instrument from left to right.

*A Few Statements in Brief.*—There are some substances used in medicine that are very injurious to teeth, and among them are the *mineral acids* which should be given with great care, largely diluted and followed quickly with water; the tinct. ferri chloridi which should scarcely be given at all, for it almost certainly not only discolors, but roughens enamel, and acts as a strong astringent on the gums, disturbing their relation with the necks of teeth. The various preparations of mercury also produce more mischief than either physicians or their patients are aware of.

It is well to be extremely cautious in ascribing pain to a particular lesion in the tooth of any pregnant female, because such pain is apt to be entirely of a neural and reflex character.

Both physician and dentist should avoid tooth extraction, if possible, where there exists the condition of pregnancy. This state, with its important interests at stake, and its prenatal influences at work, is no more to be ignored here than elsewhere. (See Dental Cosmos, Jan. No., 1879.)

In deciding upon what shall be done in the case presented, the physician will have often to take into account the disposition and prejudices of his patient. He has to deal with all grades of ignorance and obstinacy, and circumstances, perforce, will alter cases. He cannot always act up to the ideal standard which the dental specialist may set up. He may sometimes have to extract a tooth under protest, or take the chances of losing paying patronage, and most men under such circumstances will sacrifice the ideal—and the tooth.

If a person is obstinately bent on losing a tooth, he will find some one to apply the forceps if you do not. The "other fellow" will most likely rejoice in this opportunity for relieving a suffering fellow-creature, and besides take the opportunity to examine the man's tongue and prescribe for his "liver complaint." With the intelligent you can use reason, inspire confidence, and be conservative, but with some people you can't.

It may be that the city dentist, with a "select" patronage, who claims to seldom use forceps, will think I have given herein too great latitude to that instrument, but I am writing not for him, but for the country doctor; that man of many labors, who must do something of nearly everything; "in journeyings often," in perils of winds and waters, in "perils by the heathen," in the wilderness, and "among false brethren;" "in weariness and painfulness," in "watchings often," and cold and heat and storm.

In writing this I have had his interest constantly in my mind, with a view to rendering him some practical assistance. I have not suggested the purchase of anything but that I think will *pay* him, not merely in satisfaction, but pecuniarily. That pertaining to the teeth is but a small fraction of the work to which he has to give his attention, and whatever he has in the way of dental tools, and does in the line of dental work, must be limited to things simple and essential.

WENONA, ILL., Nov. 22, 1879.

